# Serum Haptoglobin as a Predictor of Treatment Response in Patients With Chronic Spontaneous Urticaria

**DOI:** 10.1002/clt2.70148

**Published:** 2026-01-07

**Authors:** Kun‐Woo Park, Boyoun Choi, Da‐Hye Moon, Se‐Min Park, Young‐Min Ye

**Affiliations:** ^1^ Department of Allergy and Clinical Immunology Ajou University School of Medicine Suwon Republic of Korea

**Keywords:** chronic spontaneous urticaria, complete control, haptoglobin, zonulin

## Abstract

**Background:**

Chronic spontaneous urticaria (CSU) is a mast cell‐driven disease associated with systemic inflammation and altered immune responses. Haptoglobin (HP), an acute‐phase glycoprotein, exhibits antioxidative and immunomodulatory properties, while zonulin, the precursor of HP‐2, regulates epithelial barrier integrity. We investigated the clinical relevance of serum HP and zonulin in CSU and their association with treatment outcomes.

**Methods:**

Serum HP and zonulin levels were measured by ELISA in 124 CSU patients and 57 healthy controls (HCs). In 62 CSU patients, follow‐up samples were obtained after 3 months of treatment. Clinical outcomes included the urticaria activity score over 7 days (UAS7) and the urticaria control test (UCT).

**Results:**

Serum HP levels were significantly higher in CSU patients than in HCs (median 1145.1 vs. 839.2 μg/mL, *p* < 0.001), whereas zonulin levels did not differ. HP correlated positively, but weakly, with the white blood cell count, C3 and C‐reactive protein (all rho ≈ 0.2), and negatively with disease duration and eosinophil percentage. Zonulin correlated negatively with UCT scores but not with HP. After treatment, HP decreased significantly (*p* < 0.001), with greater reductions in patients showing a ≥ 12‐point improvement on the UAS7. Baseline HP was higher in patients who achieved complete control (UCT = 16) than others (*p* = 0.017). ROC analysis identified baseline HP ≥ 1249 μg/mL as an optimal cutoff, confirmed as an independent predictor of complete control (odds ratio = 4.23, *p* = 0.029).

**Conclusion:**

Serum HP is elevated in CSU patients, decreases with treatment, and independently predicts complete urticaria control. HP may serve as a potentially prognostic biomarker for CSU.

## 1 Introduction

Chronic spontaneous urticaria (CSU) is a common inflammatory skin disorder characterized by recurrent wheals and/or angioedema lasting longer than 6 weeks without any identifiable external trigger [1]. In almost half of patients, autoimmunity of either type I (autoallergic) or type IIb (IgG‐mediated) contributes to persistent mast cell activation and degranulation [2, 3]. Although histamine release from mast cells and basophils is the key effector mechanism, growing evidence indicates that CSU features broader immune dysregulation, including altered cytokine networks, complement activation, and systemic inflammation [4, 5, 6, 7]. Elevated levels of inflammatory markers such as C‐reactive protein (CRP), and complement activation, have been reported, supporting the concept that CSU is not only a cutaneous disease but also a condition that includes systemic immune activation [2, 8].

Haptoglobin (HP) is an acute‐phase glycoprotein predominantly synthesized by hepatocytes in response to proinflammatory cytokines such as IL‐6 [9]. HP binds free hemoglobin, preventing oxidative tissue damage, and serves as an extracellular chaperone that stabilizes proteins under stress [10]. The antioxidant role aside, HP participates in immune regulation, modulating both lymphocyte and macrophage function. HP expression increases in patients with various inflammatory and autoimmune disorders, including systemic lupus erythematosus and rheumatoid arthritis [11, 12]. Genetic polymorphisms of the Hp2 allele that encode the precursor protein zonulin have been associated with increased susceptibility to autoimmune conditions such as type I diabetes and celiac disease [13].

Zonulin, a product of the HP‐2 precursor protein, regulates the activities of intestinal tight junctions and has been suggested to be a useful marker of epithelial barrier dysfunction [13, 14]. Increased serum zonulin levels have been associated with autoimmune and chronic inflammatory disorders, suggesting that epithelial barrier impairment triggers systemic inflammation [14]. However, any influence of zonulin on CSU remains unclear, although some have suggested that barrier alterations contribute to CSU pathogenesis. As zonulin is the precursor of HP, measurements of both proteins in parallel might reveal an interplay between systemic inflammation and barrier integrity of CSU.

In our earlier proteomic study, differential protein expression was observed between ASST‐positive and ASST‐negative patients. Notably, HP was one of seven proteins markedly upregulated in ASST‐positive patients [15], indicating a possible association with the autoimmune CSU endotype.

In this study, we analyzed serum HP and zonulin levels in CSU patients and healthy controls (HCs), explored their relationships with clinical and laboratory parameters, and evaluated the potential of both proteins as biomarkers for predicting treatment outcomes.

## 2 Materials and Methods

### 2.1 Subjects

This hospital‐based cross‐sectional study enrolled 124 patients with CSU and 57 HCs. CSU was defined when recurrent wheals and pruritus persisted for ≥ 6 weeks without any identifiable external trigger. The CSU group included 79 females and 45 males, with a median age of 38 years. The HC group comprised 35 females and 22 males, with a median age of 42 years. The two groups did not differ significantly in terms of age or sex distribution (Table 1). The exclusion criteria were any other chronic skin disease, clinically proven urticarial vasculitis, or physically induced urticaria. All participants gave written informed consent prior to enrollment. The HCs lacked any history of allergic, autoimmune, or chronic inflammatory diseases, including urticaria.

**TABLE 1:** Baseline clinical characteristics of patients with CSU and healthy controls.

Variables	CSU (*n* = 124)	NC (*n* = 57)	*p‐*value
Female sex (%)	79 (63.7)	35 (61.4)	0.869
Age (years)	38 (31–50)	42 (28–52)	0.486
BMI (kg/m^2^)	23.5 (21.3–26.7)	22.7 (21.5–24.6)	0.332
Metabolic syndrome (%)	26/122 (21.3)		
BMI ≥ 25 (%)	41 (33.1)	12 (21.1)	0.115
HTN (%)	24 (19.4)	6 (10.5)	0.196
Glucose ≥ 100 mg/dL or DM (%)	36 (29.0)		
Triglyceride ≥ 200 mg/dL (%)	56/122 (45.5)		
Low HDL^a^ (%)	22/122 (17.9)		
Asthma (%)	5 (4.0)		
Allergic rhinitis (%)	47 (37.9)		
NECU (%)	43 (34.7)		
Urticaria duration (months)	8 (4–45)		
Angioedema (%)	60/122 (49.2)		
ASST positivity (%)	76 (61.3)		
Atopy (%)	60 (48.4%)		
ANA positivity (%)	32 (25.8%)		
Total IgE (kU/L)	129 (65–233)		
Complement 3 level (mg/dL)	118 (105–134)		
Complement 4 level (mg/dL)	26 (21–32)		
WBC (10^3^/μL)	6.6 (5.6–8.4)		
Eosophils (%)	1.5 (0.8–2.5)		
Basophils (%)	0.5 (0.3–0.7)		
CRP (mg/dL)	0.08 (0.04–0.18)		
UAS7 (0–42)	21 (13–35)		
UCT score (0–16)	6 (4–9)		
Anti‐TG IgG (%)	20/52 (38.5)		
Anti‐TPO IgG (%)	12/52 (23.1)		

*Note:* Data are presented as frequencies (%) or as medians (with interquartile ranges).

Abbreviations: ANA, antinuclear antibody; ASST, autologous serum skin test; BMI, body mass index; CRP, C‐reactive protein; NECU, nonsteroidal anti‐inflammatory drug‐exacerbated chronic urticaria; TG, thyroglobulin; TPO, thyroid peroxidase; UAS7, urticaria activity score over 7 days; UCT, urticaria control test; WBC, white blood cell.

^a^ HDL < 50 mg/dL in men or < 50 mg/dL in women.

### 2.2 Clinical Assessment

Disease activity was evaluated using the Urticaria Activity Score over 7 days (UAS7) and the Urticaria Control Test (UCT) score at baseline (*n* = 124) and after 3 months of treatment (*n* = 62). Complete urticaria control was defined as a UCT score of 16 after treatment [1]. A reduction in the UAS7 ≥ 12 points from baseline indicated a minimal clinically important difference in CSU status [16].

Atopy was defined as a positive skin prick test response to at least one common inhalant allergen (pollens of alder, birch, oak, a grass mixture, mugwort, ragweed, cat or dog fur, *Dermatophagoides pteronyssinus*, *D*. *farinae*, *Aspergillus niger*, or Alternaria spp.). The levels of thyroid autoantibodies (anti‐thyroid peroxidase [TPO] and anti‐thyroglobulin [TG]) were checked. Total serum IgE levels were assayed using the ImmunoCAP system (Thermo Fisher Scientific/Phadia, Uppsala, Sweden; detection range: 2–5000 kU/L) in line with the manufacturer's instructions. The ASST was performed and interpreted as suggested by the EAACI/GA2LEN task force ASST consensus report [17].

Treatment regimens during the 3‐month follow‐up included up‐dosing H1Ahs, and add‐on omalizumab or cyclosporine, based on disease severity and physician judgment. Metabolic syndrome was assessed as a potential confounding factor using the modified National Cholesterol Education Program Adult Treatment Panel III [18]. Patients meeting ≥ 3 of the following components were classified as having metabolic syndrome: BMI ≥ 25 kg/m^2^ (reflecting the Asian adiposity threshold), Glucose ≥ 100 mg/dL or diagnosed diabetes mellitus, Blood pressure ≥ 130/85 mmHg or current use of antihypertensive medication, Triglyceride ≥ 150 mg/dL, Low HDL cholesterol (< 40 mg/dL in men, < 50 mg/dL in women).

### 2.3 Measurement of Serum Haptoglobin and Zonulin Levels

Peripheral blood samples were collected from all participants at baseline, and from 62 CSU patients after 3 months of treatment. Serum HP and zonulin levels were measured via enzyme‐linked immunosorbent assays (ELISAs) using commercial kits (R&D Systems Inc., Minneapolis, MN, USA). Prior to HP measurements (detection range: 3.1–200 μg/mL), serum samples were diluted 1:20,000. Undiluted serum samples were used when assessing zonulin levels (detection range: 0.625–40 ng/mL). At baseline sampling, no participant was on maintenance systemic corticosteroids.

### 2.4 Statistical Analysis

Data were analyzed using SPSS version 26.0 (IBM Corp., Armonk, NY, USA). Continuous variables were expressed as medians with interquartile ranges (IQRs) and categorical variables as frequencies with percentages. Between‐group comparisons employed the Mann‐Whitney U or Kruskal‐Wallis, and Fisher exact tests. The Wilcoxon signed‐rank test was used to assess within‐group changes from the baseline HP and zonulin levels after treatment. Correlations were derived using the Spearman method. Receiver operating characteristic (ROC) curves were drawn to determine the optimal baseline HP cutoff level that predicted complete urticaria control. The Youden index was employed to identify the threshold. Logistic regression analysis was used to identify independent predictors of complete control. Variables exhibiting *p*‐values < 0.05 on univariate analysis, and the baseline UAS7 and UCT score, were included in the multivariate model. A two‐sided *p*‐value < 0.05 was considered statistically significant. Graphs were prepared using GraphPad Prism version 10.0 (GraphPad Software, San Diego, CA, USA).

## 3 Results

### 3.1 Baseline Characteristics

There was with no significant between‐group difference in terms of the age or sex distribution (Table 1). The median urticaria duration of CSU patients was 8.0 months (IQR, 4.0–45.0 months). The baseline UAS7 and UCT scores in CSU patients were 21 (IQR, 13–35) and 6 (IQR, 4–9) respectively. Angioedema was apparent in 49.2% of patients, and atopy in 48.4%. In terms of treatment, 44 patients (35.5%) received add‐on therapy, including omalizumab (*n* = 30) or cyclosporine (*n* = 14). The antinuclear antibody (ANA) positivity rate, and those of anti‐TPO IgG and the ASST, were 25.8%, 23.1%, and 61.3%, respectively. The median serum total IgE level was 129 kU/L (IQR, 65–233 kU/L).

### 3.2 Serum Haptoglobin and Zonulin Levels in CSU Patients and Healthy Controls

**FIGURE 1:**
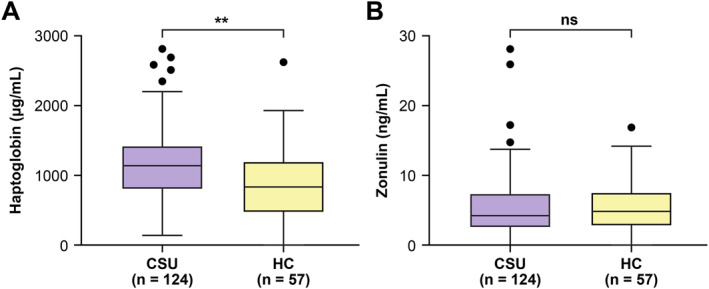
Serum haptoglobin (A) and zonulin (B) levels in CSU patients and healthy controls. **p < 0.01.

The serum HP levels were significantly higher in CSU patients (median, 1145.1 μg/mL; IQR, 798.7–1415.7 μg/mL) than HCs (839.2 μg/mL; IQR, 499.6–1189.3 μg/mL; *p* = 0.001) (Figure 1A). In contrast, the serum zonulin levels did not differ significantly between CSU patients (median, 4.4 ng/mL; IQR, 2.5–7.3 μg/mL) and HCs (4.8 ng/mL; IQR, 2.8–7.5 μg/mL; *p* = 0.579) (Figure 1B). Concurrent metabolic syndrome was found in 26 (23.1%) of 122 patients with CSU. Allergic rhinitis (*n* = 47) or asthma (*n* = 5) were present in 49 CSU patients (39.5%). NSAID‐exacerbated chronic urticaria (NECU) was identified in 43 patients (34.7%).

### 3.3 The Associations Between Serum Haptoglobin and Zonulin Levels, and Clinical and Laboratory Parameters

**FIGURE 2:**
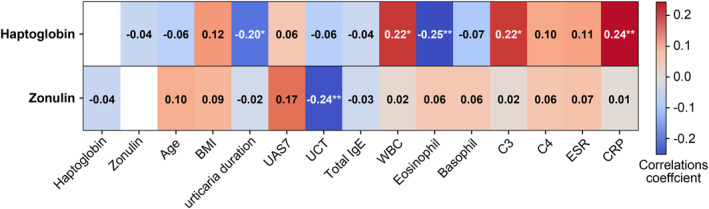
Correlations between serum haptoglobin and zonulin levels, and clinical parameters. *p < 0.05, **p < 0.01.

The baseline HP level correlated positively with the white blood cell (WBC) count (Spearman rho = 0.22, *p* = 0.015), the C3 complement level (0.22, *p* = 0.016), and the C‐reactive protein (CRP) concentration (0.24, *p* = 0.007); but negatively with disease duration (−0.20, *p* = 0.026) and the blood eosinophil percentage (−0.25, *p* = 0.006, Figure 2). The zonulin level correlated negatively with the baseline UCT score (Spearman rho = −0.24, *p* = 0.008) but not with the HP concentration. No significant difference in either the HP or zonulin level was observed by ASST‐ or ANA‐positivity or atopy status, or disease severity. Baseline HP levels were comparable between CSU patients with and without metabolic syndrome (median 1126.5 vs. 1172.1 μg/mL, *p* = 0.933), while baseline zonulin levels were significantly elevated in those meeting for metabolic syndrome (6.0 vs. 843.0 ng/mL, *p* = 0.011). Baseline HP and zonulin levels did not differ according to the presence of asthma or allergic rhinitis (HP: 1196.1 vs. 1099.6 μg/mL, *p* = 0.566; zonulin: 5.0 vs. 4.0 ng/mL, *p* = 0.413), nor according to NECU status (HP: 1196.1 vs. 1130.4 μg/mL, *p* = 0.616; zonulin: 6.0 vs. 4.0 ng/mL, *p* = 0.069).

### 3.4 Three‐Month Changes in Serum Haptoglobin and Their Associations With Clinical Improvement

**FIGURE 3:**
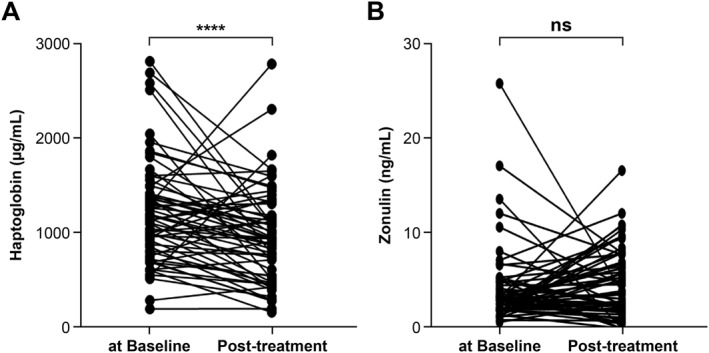
Changes in serum haptoglobin (A) and zonulin (B) levels from baseline to 3 months of treatment in 62 CSU patients.

In the 62 CSU patients for whom follow‐up data were available, the serum HP level significantly decreased after 3 months of treatment compared to baseline (*p* < 0.001, Figure 3A), whereas the serum zonulin level exhibited no significant change (Figure 3B).

Of these 62 patients, 23 (37.1%) achieved complete urticaria control (UCT = 16) after 3 months of treatment but 39 (62.9%) did not (Table 2). The baseline characteristics were comparable between the two groups in terms of age, sex, disease duration, and atopy status, as well as the baseline total IgE and ANA levels, and the ASST score. However, the body mass index (BMI) was higher and the blood eosinophil percentage significantly lower in the complete control group. The baseline UAS7 and UCT score did not differ between the groups, although the changes from baseline (both scores) were significantly greater in the complete control group. The baseline HP levels were significantly higher in patients who achieved complete control (median, 1343.8 μg/mL; IQR, 280.5–2815.0 μg/mL) than in those who did not (median, 1006.4 μg/mL; IQR, 191.7–2045.5 μg/mL; *p* = 0.017) (Figure 3A). No significant difference in the baseline zonulin level was observed between the complete and incomplete urticaria control groups.

**TABLE 2:** Univariate comparison and logistic regression analysis of predictors of complete urticaria control (UCT score = 16) at 3 months.

Variables	UCT = 16 (*n* = 20)	UCT < 16 (*n* = 42)	*p*‐value^a^	OR (95% CI)	*p* value^b^
Female sex (%)	9 (45.0)	25 (59.5)	0.413		
Age (years)	41 (39–50)	37 (28–46)	0.083		
BMI (kg/m^2^)	25.6 (23.4–28.0)	23.7 (22.3–25.2)	**0.049**	1.18 (0.96, 1.45)	0.125
Metabolic syndrome (%)	6/19 (31.6)	7/42 (16.7)	0.311		
Asthma and/or allergic rhinitis (%)	6 (30.0)	15 (37.5)	0.775		
NECU (%)	8 (40.0)	7 (16.7)	0.060		
Urticaria duration (months)	5.0 (3.5–31.0)	8.0 (3.0–36.0)	0.576		
Angioedema (%)	14 (70.0)	19 (45.2)	0.102		
ASST positivity (%)	13 (65.0)	31 (73.8)	0.554		
Atopy (%)	9 (45.0)	20 (47.6)	1.		
ANA positivity (%)	4 (20.0)	9 (21.4)	1.		
Total IgE (kU/L)	118 (5, 570)	130 (16, 1705)	0.503		
Complement 3 level (mg/dL)	126 (107–140)	120 (112–135)	0.494		
Complement 4 level (mg/dL)	29 (25–33)	26 (21–34)	0.285		
WBC (10^3^/μL)	6.8 (4.0, 13.3)	6.8 (3.5, 16.1)	0.629		
Eosinophil (%)	0.8 (0.1, 2.1)	1.4 (0, 12.3)	**0.024**	0.55 (0.24, 1.10)	0.088
Basophil (%)	0.3 (0.1, 1.1)	0.5 (0, 1.0)	0.304		
CRP (mg/dL)	0.09 (0.06–0.20)	0.08 (0.05–0.16)	0.455		
UAS7 (0–42)	26 (12–35)	19 (14–32)	0.497	1.03 (0.95, 1.11)	0.514
UCT score (0–16)	5 (3–8)	7 (4–10)	0.301	1.00 (0.79, 1.27)	0.982
H1AH refractoriness (%)	11 (55.0)	22 (52.4)	1.		
Omalizumab add‐on (%)	9 (45.0)	21 (50.0)	0.586		
Cyclosporine add‐on (%)	4 (20.0)	10 (23.8)	1.		
Anti‐TPO IgG (%)	4/10 (40.0)	5/19 (26.3)	1.		
Zonulin (ng/mL)	3.1 (2.2–6.8)	3.1 (1.9–4.0)	0.166		
Haptoglobin (μg/mL)	1343.8 (1112.9–1902.0)	1006.4 (750.6–1369.4)	**0.017**		
Haptoglobin ≥ 1249 μg/mL	14 (70.0)	13 (31.0)	**0.006**	5.03 (1.35, 18.8)	**0.016**
CFB in haptoglobin	−306.1 (−515.3 to −60.6)	−286.2 (−443.5 to 50.7)	0.448		
CFB in zonulin	−0.5 (−4.1 to 1.7)	−0.3 (−2.9 to 1.4)	0.988		
CFB in UAS7	−25.5 (−35 to −12)	−12.0 (−20 to −6)	**0.005**		
CFB in UCT	11.5 (8–13)	5.0 (3–8)	**< 0.001**		

*Note*: Median (interquartile range). Bold values indicate statistically significant differences (*p* < 0.05).

Abbreviations: ANA, antinuclear antibody; ASST, autologous serum skin test; BMI, body mass index; CFB, change from baseline; CRP, C‐reactive protein; H1AH, H1‐antihistamine; NECU, nonsteroidal anti‐inflammatory drug‐exacerbated chronic urticaria; TPO, thyroid peroxidase; UAS7, urticaria activity score over 7 days; UCT, urticaria control test; WBC, white blood cell.

^a^ Fisher's exact test or the Mann‐Whitney *U*‐test.

^b^ Logistic regression.

Treatment regimens did not differ between complete and incomplete control groups (omalizumab add‐on: 45.0% vs. 50.0%, cyclosporine add‐on: 20.0% vs. 23.8%, Table 2). Furthermore, when patients were stratified by treatment type (H1AH updosing, omalizumab add‐on, cyclosporin add‐on), baseline HP levels (median 1115 vs. 1144.0 vs. 1215.9 μg/mL, *p* = 0.944), 3‐month HP levels (1043.5 vs. 929.7 vs. 816.3 μg/mL, *p* = 0.592), and changes from baseline (−143.5 vs. −312.5 vs. −332.0 μg/mL, *p* = 0.688) were not significantly different across treatment groups.

Changes from baseline in both HP (median −37.4 vs. −332.0 μg/mL, *p* = 0.224) and zonulin (−0.1 vs. −0.5 ng/mL, *p* = 0.192) levels did not differ between patients with and without metabolic syndrome. The proportion of patients achieving complete control was also comparable between the two groups (46.2% vs. 27.1%, *p* = 0.311). In addition, the proportion of patients with comorbid asthma/rhinitis (30.0% vs. 37.5%, *p* = 0.775) and NECU (40.0% vs. 16.7%, *p* = 0.060) were similar between the complete‐control and incomplete‐control groups.

Using the UCT ≥ 12 threshold to define well‐controlled urticaria, 48 of 62 patients (77.4%) met this criterion after 3 months of treatment (Supporting Information Table S1). Baseline HP levels were significantly higher in the well‐controlled group than in those with UCT < 12 (median 1215.9 vs. 870.6 μg/mL, *p* = 0.031). HP reduction was also significantly greater in patients achieving UCT ≥ 12 (−339.0 vs. + 51.5 μg/mL, *p* = 0.013).

When a clinical response was defined by a UAS7 ≥ 12 points, such patients exhibited a more pronounced reduction in HP level after treatment compared to non‐responders (median change, −351.5 vs. −93.6 μg/mL; *p* = 0.021). In contrast, the zonulin levels showed no significant change after treatment in either response group.

To further explore whether individual patterns of HP change were associated with treatment response, patients were categorized into HP decrease (≥ 20% from baseline, *n* = 35, HP stable < 20% change, *n* = 18), and HP increase (≥ 20% from baseline, *n* = 9). Comparative analysis was performed between HP decrease groups and the combined HP stable/increase group. The proportions of patients achieving UCT = 16 (31.4% vs. 33.3%, *p* = 1.000) and UCT ≥ 12 (88.6% vs. 66.7%, *p* = 0.058) did not differ significantly. However, a clinically meaningful UAS7 reduction (≥ 12 points) was more frequently observed in the HP decrease group (74.3% vs. 40.7%, *p* = 0.010).

### 3.5 Predictors of Complete Urticaria Control

ROC analysis indicated that a baseline HP level ≥ 1249 μg/mL may help identify patients more likely to achieve complete urticaria control (AUC = 0.69, 95% CI = 0.55–0.83; sensitivity, 65.2%; specificity, 74.4%; *p* = 0.007). In the multivariate model adjusting for baseline UAS7 and UCT scores, BMI, and blood eosinophil percentage, a baseline HP ≥ 1249 μg/mL remained statistically significant as an independent predictor of complete urticaria control (adjusted odds ratio, 4.23; 95% CI, 1.16–15.46; *p* = 0.029, Table 2). In contrast, this cutoff did not predict achievement of well‐controlled disease (Supporting Information Table S1).

## 4 Discussion

This study showed that serum HP levels were significantly elevated in CSU patients compared to HCs, but the zonulin levels did not differ between the groups. Notably, the HP levels decreased after 3 months of treatment, and changes from baseline were associated with clinically meaningful improvements in the UAS7 and UCT score. Furthermore, a baseline HP ≥ 1249 μg/mL predicted complete urticaria control after 3 months of treatment, as confirmed by multivariate analysis.

These results extend previous evidence that CSU is accompanied by systemic inflammation. Earlier studies reported increased levels of CRP, IL‐6, and complement components in CSU patients, underscoring the involvement of acute‐phase reactants in the disease [19, 20]. HP, a classical acute‐phase protein induced by IL‐6, has not been extensively studied in the CSU context. In our earlier proteomic analysis, HP was among proteins upregulated in the sera of ASST‐positive patients, suggesting a possible link with autoimmune CSU endotypes [15]. However, in the present study, the HP levels did not differ by ASST, ANA, or thyroid autoantibody status, indicating that the elevation more likely reflects systemic inflammatory activity rather than an autoimmune mechanism per se. Moreover, although HP correlated with several inflammatory markers such as CRP and C3, the correlation coefficients were weak (rho ≈ 0.2), suggesting that HP captures a broader and only partially overlapping dimension of systemic inflammation rather than directly mirroring classical acute‐phase reactants.

Raynes et al. [21] reported that the HP level rose during the acute‐phase response, linking inflammatory processes to adaptive immune regulation. In line with this, our data indicate that the HP level was elevated in CSU patients and fell dramatically in those who achieved complete control after treatment, supporting a role for HP as a marker of both inflammation and disease resolution. HP scavenges free hemoglobin and exerts antioxidative effects, and also serves as an immune modulator. Arredouani et al. [11] found that HP directly suppressed T‐helper 2 cell cytokine release by acting on T cells suggesting that HP plays a regulatory role in allergic inflammation. Beyond these previously described functions, HP may also influence mast‐cell‐related inflammation indirectly. Although HP is not known to directly activate or inhibit mast cells, its upstream regulation by IL‐6 and its capacity to reduce oxidative stress, an enhancer of mast‐cell activation, suggest that it reflects cytokine activity relevant to mast‐cell responsiveness. Collectively, these mechanistic links suggest that imply that HP acts not only as a nonspecific acute‐phase reactant but also as a potential immunomodulatory molecule integrating signals from cytokine‐drive and T‐cell‐mediated pathways that may contribute to CSU pathophysiology.

Zonulin, the precursor of HP‐2, is a regulator of epithelial tight junctions and has been implicated in the barrier dysfunction of patients with autoimmune and chronic inflammatory diseases [13]. Elevated serum zonulin levels have been reported in patients with severe atopic dermatitis [22] and severe asthma [23]. Prior studies on CSU, however, have yielded inconsistent results. Ünal et al. [24] found significantly increased zonulin levels in CSU patients, especially in those with angioedema, but Kamal et al. [25] reported lower zonulin levels in CSU patients than others and no association with the UAS7 or the total IgE level. In our cohort, the zonulin levels of patients did not differ significantly from those of controls and exhibited no treatment‐related changes, although the baseline zonulin level correlated negatively with the UCT score, suggesting a potential link to disease control rather than disease activity. The among‐study differences may reflect methodological variations, distinct population characteristics, or disease heterogeneity. Importantly, our results indicate that zonulin may not be a consistent systemic biomarker of CSU across populations, in contrast to HP, an elevated level of which was significantly predictive. Moreover, in our earlier study, we observed increased expression of the epidermal barrier protein filaggrin in CSU lesions, correlating with disease severity, whereas filaggrin was downregulated in the skin of those with atopic dermatitis [26]. This suggests that CSU may involve a barrier physiology that differs from that of other allergic diseases.

The clinical relevance of HP in the CSU context lies is the association thereof with treatment outcomes. Patients who achieved complete urticaria control had higher baseline HP levels than those exhibiting incomplete control, and the reductions in HP levels after treatment paralleled improvements in the UAS7 and UCT score. Notably, a baseline HP level ≥ 1249 μg/mL independently predicted complete urticaria control. The predictive performance was comparable to those of other proposed prognostic markers such as the total IgE, CRP, or D‐dimer level. This cutoff value may be clinically useful when stratifying patients and anticipating treatment trajectories, although validation in larger multicenter cohorts is required. Consistent with these findings, baseline HP also predicted achievement of well‐controlled urticaria (UCT ≥ 12); patients attaining this outcome exhibited higher baseline HP levels and greater reductions after treatment. However, changes in HP did not discriminate patients with complete control, indicating that HP dynamics primarily reflect the degree of inflammatory resolution rather than functioning as an independent determinant of complete control.

Importantly, HP levels were not influenced by treatment modality. Baseline, post‐treatment, and change‐from‐baseline HP values did not differ among patients treated with updosed H1AH or add‐on therapies with omalizumab or cyclosporine. Likewise, the distribution of treatment types was similar between patients who achieved complete control and those who did not, supporting the interpretation that HP reflects underlying inflammatory burden rather than drug‐specific effects.

We also assessed potential confounders. Baseline HP and changes after treatment did not differ according to metabolic syndrome status, autoimmune markers, or inflammatory comorbidities. Respiratory allergic comorbidities and NECU also did not influence HP or zonulin levels, nor did they affect the likelihood of achieving complete control. Together, these analyses support that HP elevation in CSU is less likely driven by metabolic, autoimmune, or allergic comorbidities, and instead more plausibly reflects CSU‐related systemic inflammation.

Several limitations of this work should be acknowledged. First, this was a single‐center study with a modest sample size, particularly of the longitudinal subgroup, which may limit generalizability. The discriminative performance of baseline HP was moderate, and therefore the proposed cutoff value should be interpreted as exploratory. Second, the treatment regimens were heterogeneous, precluding firm conclusions about drug‐specific effects. Third, mechanistic studies were not performed. It remains unclear, therefore, whether HP is only a marker of systemic inflammation or an active contributor to CSU pathogenesis.

In conclusion, our study highlights the fact that the serum HP level may serve as a potential CSU biomarker. Elevated HP reflects systemic inflammation, decreases with treatment, and independently predicts complete urticaria control. In contrast, zonulin does not appear to play a major role in CSU pathogenesis. Although these findings provide clinically meaningful insights, they should be interpreted cautiously and require validation in larger, multicenter and mechanistic studies to clarify the utility of HP in guiding treatment strategies and monitoring outcomes in CSU.

## Author Contributions

**Kun‐Woo Park:** methodology, writing – original draft, investigation, formal analysis. **Boyoun Choi:** methodology, investigation, data curation. **Da‐Hye Moon:** investigation, methodology. **Se‐Min Park:** investigation, methodology. **Young‐Min Ye:** conceptualization, investigation, formal analysis, data curation, supervision, project administration, funding acquisition, writing – review and editing, writing – original draft, resources.

## Funding

This work was supported by grants from the National Research Foundation of Korea funded by the Korean government (NRF‐2022R1A2C2006607) and the GRRC program of Gyeonggi province (GRRCAjou2023‐B01).

## Ethics Statement

Written informed consent was obtained from all subjects. Biospecimens from and data on healthy subjects were provided by the Biobank of Ajou University Hospital, which is a member of the Korea Biobank Network. The study was approved by the Institutional Review Board of Ajou University Hospital (AJOUIRB‐KSP‐2021–075).

## Conflicts of Interest

The authors declare no conflicts of interest.

## Supporting information


Supporting Information S1

## Data Availability

The data that support the findings of this study are available from the corresponding author upon reasonable request.
