# Supplementary material for: Serum Haptoglobin as a Predictor of Treatment Response in Patients With Chronic Spontaneous Urticaria
Source: Clin Transl Allergy. 2026 Jan 7;16(1):e70148. doi: 10.1002/clt2.70148 (PMC12777545; doi:10.1002/clt2.70148)
Supplement: Supplementary file 1 — Supporting Information S1 [file CLT2-16-e70148-s001.docx]

Supplementary Table S1. Univariate comparison and logistic regression analysis of predictors of well-controlled urticaria (UCT ≥ 12) at 3 months.

| Variables | UCT ≥ 12  (n = 48) | UCT < 12  (n = 14) | *P*-value* | OR (95% CI) | P value^†^ |
| --- | --- | --- | --- | --- | --- |
| Female sex (%) | 25 (52.1) | 9 (64.3) | 0.546 |  |  |
| Age (years) | 39 (32, 46) | 42 (29, 57) | 0.464 |  |  |
| BMI (kg/m^2^) | 24.1 (22.4, 26.7) | 24.7 (22.5, 27.2) | 0.711 |  |  |
| Metabolic syndrome (%) | 9/47 (19.1) | 4 (28.6) | 0.472 |  |  |
| Asthma and/or Allergic rhinitis (%) | 15 (31.3) | 6 (46.2) | 0.349 |  |  |
| NECU (%) | 15 (31.3) | 0 | **0.014** | 0 | 0.998 |
| Urticaria duration (months) | 7.5 (4.0, 42.0) | 4.5 (2.0, 36.0) | 0.303 |  |  |
| Angioedema (%) | 29 (60.4) | 4 (28.6) | 0.066 |  |  |
| ASST positivity (%) | 33 (68.8) | 11 (78.6) | 0.739 |  |  |
| Atopy (%) | 23 (47.9) | 6 (42.9) | 0.771 |  |  |
| ANA positivity (%) | 11 (22.9) | 2 (14.3) | 0.713 |  |  |
| Total IgE (kU/L) | 126 (67, 230) | 135 (52, 264) | 0.960 |  |  |
| Complement 3 (mg/dL) | 122 (110, 138) | 120 (112, 135) | 0.805 |  |  |
| Complement 4 (mg/dL) | 28 (22, 33) | 26 (21, 35) | 0.958 |  |  |
| WBC (10^3^/µL) | 7.5 (5.6, 9.3) | 6.6 (5.0, 7.6) | 0.236 |  |  |
| Eosinophil (%) | 1.0 (0.6, 1.8) | 2.3 (1.2, 3.9) | **0.003** | 0.90 (0.63, 1.28) | 0.552 |
| Basophil (%) | 0.4 (0.2, 0.6) | 0.7 (0.4, 0.9) | **0.004** | 0.02 (0.00, 1.04) | 0.053 |
| CRP (mg/dL) | 0.08 (0.05, 0.20) | 0.07 (0.05, 0.10) | 0.274 |  |  |
| UAS7 (0-42) | 19 (14, 32) | 23 (13, 35) | 0.742 | 1.05 (0.95, 1.17) | 0.366 |
| UCT score (0-16) | 7 (4, 10) | 4 (2, 8) | 0.171 | 1.49 (1.06, 2.10) | **0.020** |
| H1AH refractoriness (%) | 27 (56.3) | 6 (42.9) | 0.544 |  |  |
| Omalizumab add-on (%) | 27 (56.3) | 8 (57.1) | 1.000 |  |  |
| Cyclosporine add-on (%) | 10 (20.8) | 4 (28.6) | 0.717 |  |  |
| Anti-TPO IgG (%) | 8/22 (36.4) | 1/7 (14.3) | 0.382 |  |  |
| Zonulin (ng/mL) | 3.0 (2.1, 4.3) | 3.1 (1.9, 4.9) | 0.686 |  |  |
| Haptoglobin (µg/mL) | 1,215.9 (923.0, 1,5282.5) | 870.6 (608.0, 1,299.8) | **0.031** | 1.00 (1.00, 1.01) | **0.023** |
| Haptoglobin ≥ 1,249 µg/mL | 23 (47.9) | 4 (28.6) | 0.235 |  |  |
| CFB in haptoglobin | –339.0 (–535.8, –102.9) | 51.5 (–331.3, 363.2) | **0.013** |  |  |
| CFB in zonulin | –0.2 (–3.1, 1.8) | –1.1 (–5.0, 0.4) | 0.304 |  |  |
| CFB in UAS7 | –16 (–28, –8) | –10 (–17, 3) | **0.019** |  |  |
| CFB in UCT | 11.5 (8, 13) | 5.0 (3, 8) | **0.003** |  |  |

Median (interquartile range), *Fisher’s exact test or the Mann-Whitney U-test, ^†^Logistic regression

BMI, body mass index; NECU, nonsteroidal anti-inflammatory drug-exacerbated chronic urticaria; ASST, autologous serum skin test; ANA, antinuclear antibody; WBC, white blood cell; CRP, C-reactive protein; UAS7, urticaria activity score over 7 days; UCT, urticaria control test; H1AH, H1-antihistamine; TPO, thyroid peroxidase; CFB, change from baseline
